# Environmental and vegetation controls on the spatial variability of CH_4_ emission from wet-sedge and tussock tundra ecosystems in the Arctic

**DOI:** 10.1007/s11104-014-2377-1

**Published:** 2015-01-11

**Authors:** Katherine Rose McEwing, James Paul Fisher, Donatella Zona

**Affiliations:** 1 Department of Animal and Plant Science, University of Sheffield, Western Bank, Sheffield S10 2TN UK; 2 Department of Biology, San Diego State University, 5500 Campanile Drive, San Diego, CA 92182 USA

**Keywords:** Arctic, Climate change, Permafrost, Greenhouse gas emission, Vegetation control

## Abstract

**Aims:**

Despite multiple studies investigating the environmental controls on CH_4_ fluxes from arctic tundra ecosystems, the high spatial variability of CH_4_ emissions is not fully understood. This makes the upscaling of CH_4_ fluxes from plot to regional scale, particularly challenging. The goal of this study is to refine our knowledge of the spatial variability and controls on CH_4_ emission from tundra ecosystems.

**Methods:**

CH_4_ fluxes were measured in four sites across a variety of wet-sedge and tussock tundra ecosystems in Alaska using chambers and a Los Gatos CO_2_ and CH_4_ gas analyser.

**Results:**

All sites were found to be sources of CH_4_, with northern sites (in Barrow) showing similar CH_4_ emission rates to the southernmost site (ca. 300 km south, Ivotuk). Gross primary productivity (GPP), water level and soil temperature were the most important environmental controls on CH_4_ emission. Greater vascular plant cover was linked with higher CH_4_ emission, but this increased emission with increased vascular plant cover was much higher (86 %) in the drier sites, than the wettest sites (30 %), suggesting that transport and/or substrate availability were crucial limiting factors for CH_4_ emission in these tundra ecosystems.

**Conclusions:**

Overall, this study provides an increased understanding of the fine scale spatial controls on CH_4_ flux, in particular the key role that plant cover and GPP play in enhancing CH_4_ emissions from tundra soils.

## Introduction

Global warming in the Arctic is occurring at nearly twice the global average rate (IPCC 2013), resulting in increased temperatures, permafrost degradation, decreased snow-cover duration, changes in the hydrological cycle and changes in vegetation composition (Callaghan et al. 2010; Hinzman et al. 2005, 2013; IPCC 2013). Warmer temperatures may stimulate increased release of carbon dioxide (CO_2_) and methane (CH_4_) from tundra ecosystems (Billings et al. 1982; von Fischer et al. 2010; Harazono et al. 2006; Oechel et al. 1995; Zona et al. 2009) which are largely temperature and moisture limited. The global warming potential (GWP_100_) of CH_4_ is 28.5 times greater than that of CO_2_, making it an important greenhouse gas (IPCC 2013). CH_4_ concentration increased in the Arctic by 31 % between 2003 and 2007 accounting for around 8–10 % of global CH_4_ emissions (Bloom et al. 2010; Dlugokencky et al. 2011). In addition to temperature, the hydrological status of the soil is a very important control on CH_4_ fluxes (Bubier et al. 1993; Moore and Roulet 1993; Zona et al. 2009). The predicted increase in rainfall at northern high latitudes (IPCC 2013) may increase CH_4_ loss by increasing the anoxic status of the soil (Bhullar et al. 2013b; Blodau et al. 2004; Moore and Roulet 1993; Sebacher et al. 1986). Finally, as vegetation has a significant role for both CH_4_ transport and for the provision of substrate for methanogens, vegetation changes might significantly affect the Arctic CH_4_ budget (Bhullar et al. 2013a; Joabsson and Christensen 2001; Shannon et al. 1996; Walter and Heimann 2000).

The processes controlling methanogenesis are tightly coupled to surrounding environmental conditions (von Fischer et al. 2010; Harazono et al. 2006; Harriss and Frolking 1992; Jones et al. 1987) and are holocoenotic (Billings 1952). Because of the complexity of arctic ecosystems, there are still large uncertainties in the impact that environmental changes will have on CH_4_ emissions from the Arctic, with different CH_4_ models disagreeing on both the direction and magnitude of future changes in CH_4_ emissions from northern high latitudes with warming and increased CO_2_ (Melton et al. 2013).

Production, oxidation and transport are the three most important processes in controlling the rate of arctic tundra CH_4_ emission (Brummell et al. 2012; Bubier et al. 1993; Cao et al. 1996; von Fischer et al. 2010; Harazono et al. 2006; Lai 2009). CH_4_ is transported from the soil to the atmosphere through four main pathways: it can diffuse directly across the surface of the soil, be transported by pressure changes and wind, released as bubbles of gas (ebullition) in standing water (Bubier et al. 1993; Klapstein et al. 2014; Walter et al. 2006) or it can diffuse through the aerenchyma of vascular plants (Joabsson et al. 1999; Whalen and Reeburgh 1992). Therefore changes in vegetation composition and density might also substantially impact CH_4_ emissions (Lai et al. 2014a,b; Sebacher et al. 1985; Shannon et al. 1996). Vegetation can have a key influence on CH_4_ fluxes (von Fischer and Hedin 2007; Harazono et al. 2006; Jones et al. 1987; Schimel 1995; Ström et al. 2003) through the supply of organic substrates for CH_4_ production and by increasing CH_4_ transport from the soil to the atmosphere (Bhullar et al. 2013a; Joabsson and Christensen 2001; Noyce et al. 2014; Schimel 1995; Shannon et al. 1996; Torn and Chapin 1993). Photosynthetically driven root exudation of organic compounds and the decomposition of dead plant matter provides the primary substrates for CH_4_ production (Joabsson et al. 1999; King et al. 1998; Lai 2009; Olefeldt et al. 2013; Shannon et al. 1996; Singh 2001; Ström et al. 2012). Post production, plants facilitate transport of CH_4_ by providing important conduits for CH_4_ flux between the soil and atmosphere (Bhullar et al. 2013a,b; Brummell et al. 2012; Joabsson et al. 1999; Ström et al. 2003; Whalen 2005), allowing CH_4_ to bypass oxic layers within the soil where it would otherwise be re-oxidised (Frenzel and Karofeld 2000; Heilman and Carlton 2001; Inubushi et al. 2001; Jespersen et al. 1998; Joabsson and Christensen 2001; Ström et al. 2005; Whalen and Reeburgh 1990; Wilson and Humphreys 2010). Structurally, the tissue of some vascular plants found in tundra, especially sedges, are comprised of soft aerenchyma and lacunae tissues which contain tiny airspaces that allow for this gaseous exchange between roots and shoots via molecular diffusion (Armstrong and Armstrong 1991; Le Mer and Roger 2001; Shannon et al. 1996; Torn and Chapin 1993). The importance of vascular plants in CH_4_ emission is particularly evident during the growing season when the increase in the plant productivity and plant biomass, by increasing both substrate availability and the CH_4_ transport, ultimately increases CH_4_ emissions (Couwenberg et al. 2011; von Fisher and Hedin 2007; Greenup et al. 2000; Grunfeld and Brix 1999; Joabsson et al. 1999; Joabsson and Christensen 2001; Shannon et al. 1996). On the other hand, vascular plants can aid the competing process of CH_4_ oxidation by transporting O_2_ to their roots which supports methanotrophy when it is released to the surrounding soil (Conrad 1996; Harazono et al. 2006; Sebacher et al. 1985). The net effect of these processes helps determine the CH_4_ emissions from arctic ecosystems (Harazono et al. 2006; Joabsson et al. 1999; Shannon et al. 1996). Increased CH_4_ emission has been found to correlate with higher abundances of more conductive vascular plant species such as graminoids (Bhullar et al. 2013a,b; Bubier et al. 1993; Dias et al. 2010; Ström et al. 2003, 2005).

The complexity and heterogeneous pattern of all these biotic and abiotic processes controlling CH_4_ fluxes leads to high variations in CH_4_ measurements across arctic landscapes, as measured by chamber flux and eddy covariance techniques (Budishchev et al. 2014; Kutzbach et al. 2004; Morrissey and Livingston 1992; Sebacher et al. 1986). For example, previously reported cumulative peak growing season rates (late July to August) range from 30 to 120 mg C CH_4_ m^−2^ d^−1^ with daily averages ranging from 4.5 to 9.6 mg C CH_4_ m^−2^ d^−1^ and can vary considerably, even across consecutive measurements within the same sites (Harazono et al. 2006; Sturtevant and Oechel 2013; Vourlitis and Oechel 1997; Whiting and Chanton 1993; Wille et al. 2008). Despite extensive research into the patterns and controls of CH_4_ emissions from the Arctic (Joabsson et al. 1999; Lai et al. 2014a,b; Morrissey and Livingston 1992; Schimel 1995; Sturtevant and Oechel 2013; Whalen and Reeburgh 1990; Zona et al. 2009) the most important limiting factors, their relative importance, and the role of vegetation in controlling CH_4_ emissions are still highly debated. Some studies have argued that methanogenesis (and overall CH_4_ emissions from the Arctic) is substrate limited (Dunfield et al. 1993; King et al. 2002; Rinnan et al. 2007; Ström et al. 2003; Yoshitake et al. 2007) while others identify transport as the key limitation for CH_4_ emission (Bhullar et al. 2013a; Joabsson et al. 1999; Joabsson and Christensen 2001; Schimel 1995; Sebacher et al. 1985). To add further complexity, vegetative and environmental controls driving CH_4_ exchange within the tundra ecosystem are not independent, but rather have a combined influence upon local CH_4_ flux. For example, differences in water table levels, soil temperatures, pH and nutrient content not only directly affect CH_4_ production within the soil, but also determine the growth rates, activities and compositions of vascular plants, thus indirectly influencing vegetation control of CH_4_ fluxes (Couwenberg et al. 2011; von Fischer et al. 2010; Harazono et al. 2006; Lai et al. 2014a,b; Schimel 1995).

To enhance our understanding of these complex controls on CH_4_ emission, we measured CH_4_ fluxes using portable chambers across four arctic tundra ecosystems, including wet-sedge tundra and tussock tundra ecosystems, with different degrees of polygonization. Portable chamber measurements of microrelief patterns in greenhouse gas fluxes are useful for disentangling the fine scale environmental and vegetation controls on CH_4_ emission and will provide a basis for upscalling to generate estimates of CH_4_ flux patterning at the ecosystem scale (Hill et al. 2009; Sachs et al. 2010).

In order to determine the relative importance of environmental controls on CH_4_ flux, an extensive range of environmental variables were measured alongside CH_4_ fluxes, together with a classification of vegetation types, in these four sites in Alaska. Net ecosystem exchange (NEE), ecosystem respiration (ER) and gross primary productivity (GPP) were also determined to assess the importance of plant productivity on CH_4_ emissions. We hypothesised that increased soil and air temperature, water table height, vascular plant cover, GPP and thaw depth would be associated with increased CH_4_ emissions. We also expected that interactions between these factors may be important in determining rates of CH_4_ flux.

## Methods

### Site description

This study was performed at four sites: three in the northern part of the Arctic Coastal Plain (Barrow) (BEN, 71°17ʹ11.80ʺN, 156°36ʹ12.23ʺW, BES, 71°16ʹ51.17ʺN, 156°35ʹ47.28″W and BEO, 71° 16ʹ 51.61ʺN, 156° 36ʹ 44.44ʺ W) (Zona et al. 2009, 2012) and one at the foothill of the Brooks Range (Ivotuk, 68.49° N, 155.74° W). The Barrow study sites (BEN, BEO, and BES) are located in the North Coast of Alaska, USA. The vegetation in these northern sites is classified as sedge-moss wetland (CAVM Team 2003; W2, Walker et al. 2005), and includes prostrate dwarf shrubs, lichen, grass, forbs, rushes and bryophytes (CAVM Team 2003; Raynolds et al. 2005; Walker et al. 2005); with substantial ice wedge polygon development (Billings and Peterson 1980; Britton 1957). The presence of permafrost and the substantial development of ice-wedge polygons results in large spatial heterogeneity with high and dry oxic rims and low anoxic centres, with high water tables for most of the growing season (Harazono et al. 2006; Kwon et al. 2006; Vourlitis and Oechel 1997; Zona et al. 2009, 2011). High environmental microtopographic variation allows colonisation by a wide variety of moss, lichen and vascular dwarf shrub vegetation (Billings and Peterson 1980). Among these three study sites, BEN and BEO have the more developed polygons (low-centre and high-centre polygons respectively), while the BES site presents fairly flat and homogenous terrain. The southern study location (Ivotuk), is classified as tussock-sedge, dwarf-shrub, moss tundra and has no substantial polygon development (Riedel et al. 2005; Romanovsky et al. 2003; G4, Walker et al. 2005).

**Fig. 1:**
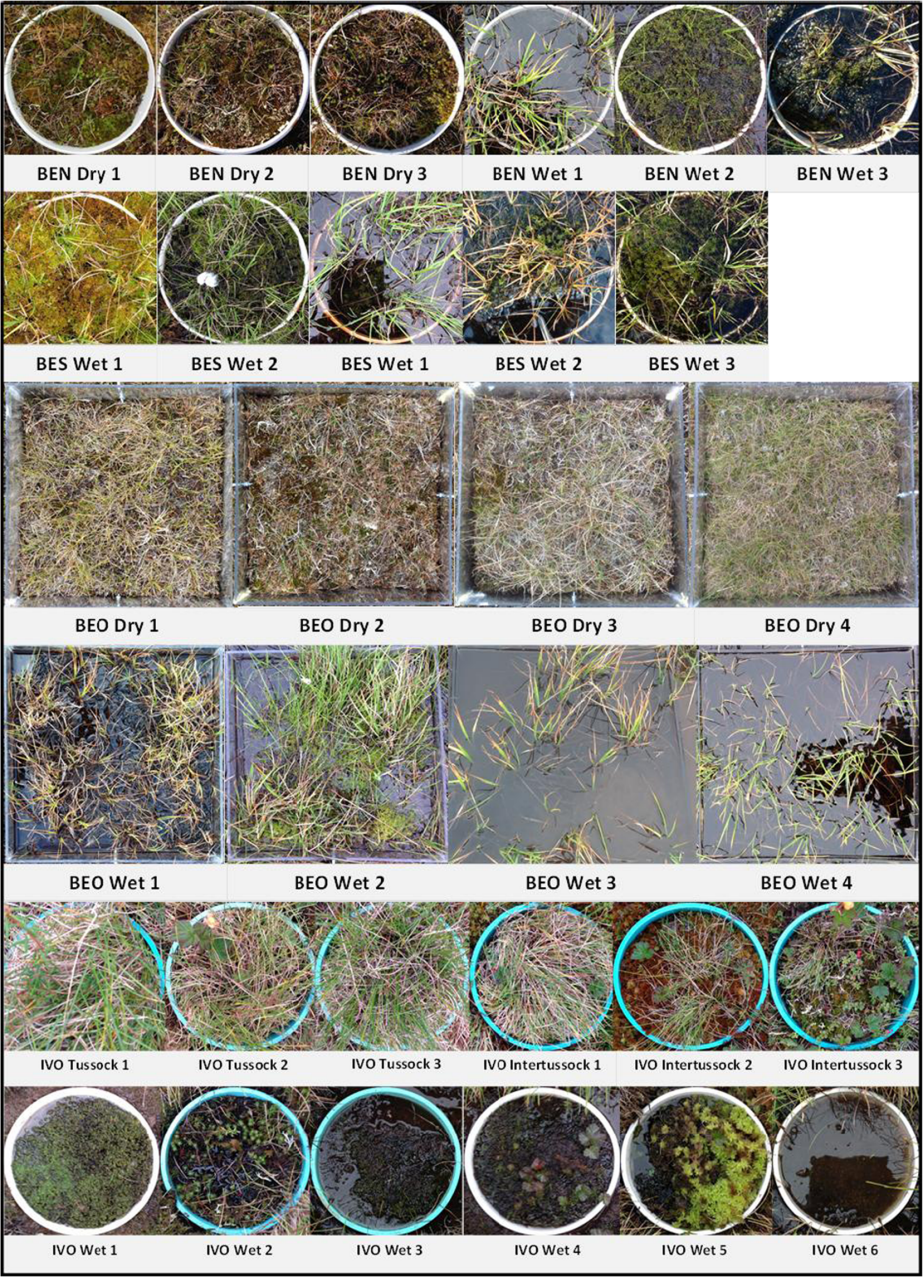
Soil collar vegetation sites at Barrow; BEN (low-centre, developed polygons), BEO (high-centre, developed polygons) and BES (fairly flat, homogenous terrain) and Ivotuk (IVO) (no substantial polygon development); From *top* to *bottom* row: three dry and three wet sites in BEN; two dry and three wet sites in BES; four dry sites in BEO; four wet sites in BEO; three tussock and three intertussock dry sites in IVO; six wet sites in IVO

The multiple sampling locations in Barrow included a variety of microhabitats with different local environmental conditions and vegetation types. In the BEO site at the beginning of the summer, eight colourless transparent acrylic soil collars (200 x 440 x 440 mm) were inserted into the moss layer with a serrated knife. These eight sampling plots were located across a 100 m transect including drier polygon rims (dry sites) and wetter polygons centres (wet sites), spaced approximately 5–10 m apart. Sites were classified as wet when the water table was at or above the soil surface level for the entire duration of the measuring period (soils were assumed to be mostly anaerobic for the entire summer); dry sites presented water tables below surface level (1 cm or deeper) for the entire measuring period (therefore containing an upper oxic soil layer, where CH_4_ oxidation can potentially occur). The cylindrical collars (radius 140 mm) used in the BES and BEN sites were inserted during a previous experiment in summer 2005 (Zona et al. 2009). Finally, in Ivotuk, cylindrical collars (radius 100 mm) were inserted, using the serrated knife method, in six wet sites and six dry sites (where the dry sites comprised of three tussock sites and three inter-tussock sites) (Fig. 1). Across all these four sites in both Barrow and Ivotuk, there were 15 sites with water table permanently below the surface (dry sites) and 16 sites flooded for the entire summer (wet sites) (Fig. 1). All soil collars were left for 24 h before measurements began to avoid soil disturbance effects on trace gas flux measurements.

### CH4 and CO2 flux measurements

All sites in Barrow were measured between the end of July and the beginning of September 2013. CH_4_ and CO_2_ fluxes were measured on a weekly basis for six weeks in Barrow (29th July to 15th September 2013) and once in Ivotuk (18th August 2013). The Barrow sites are within driving distance from a research station, which allowed multiple sampling during the season, while the remote location of Ivotuk, with no commercial airport, required chartering a plane and was accessed only once during the summer. CH_4_ and CO_2_ fluxes at each site were measured using an LGR™, Ultra-Portable Greenhouse Gas Analyser (Model 915–0011, Los Gatos Research, Palo Alto, CA, USA) with a 1 Hz sampling rate, connected to a transparent, colourless acrylic chamber. At BEO, the large clear acrylic chamber (638 × 440 × 440 mm) was connected via inlet and outlet tubing (3.5 m by 2 mm internal diameter of Bev-A-Line) to the LGR™ analyser. An elastic bungee rope was attached between the chamber and collar to ensure a gas tight seal (Moosavi and Crill 1997). At BES and BEN, smaller cylindrical chambers (140 mm height x 290 mm diameter) were used. Sampling at Ivotuk was performed using an opaque Licor (LI-8100A) automated soil CO_2_ Flux System (155 height x 188 mm diameter) clamped closed, and connected to the LGR™ to collect gas fluxes under respiratory conditions for 2.5 min. Similar sized chambers were used in previous studies at these sites (Oberbauer et al. 2007; Olivas et al. 2011; Vourlitis et al. 1993; Zona et al. 2011) and their fluxes were in close agreement with fluxes estimated by eddy covariance, despite the difference in size (Oechel et al. 1998; Zona et al. 2011).

Before each measurement, the chamber was carefully placed on each collar forming a gas tight seal. At BEO, the chamber was left on each soil collar for 4.5 min to achieve a stable increase in CH_4_ and CO_2_ concentration within the chamber headspace. The chamber was then lifted from the collar and waived in the air to expel any built up gas and to allow for ambient air levels to re-establish. The chamber was then covered with a black felt blanket and placed back on the collar for an additional 4.5 min to measure ER and estimate GPP (GPP = NEE + ER). As the CH_4_ fluxes did not differ between the dark and light measurements, means of these values were used to perform the statistical analysis. Because of the smaller size of the chamber used in BEN and BES, and the shorter time required to achieve a stable increase in CO_2_ and CH_4_ concentration, both light and dark measurements in these two sites were performed for 2.5 min each.

CH_4_ and CO_2_ fluxes were calculated from the linear increase in gas concentrations inside the chamber headspace as measured by the LGR™. Least squares linear regression was applied to the increase in CH_4_ after chamber closure. The obtained rate of concentration increase was then used with the following equation to obtain the CH_4_ and CO_2_ flux at each site.

Where:

$$ {F}_0=S\frac{V\;M\;273.16}{A{V}_m\left(273.16+T\right)}3600 $$

F_0_: Flux at the time of chamber closure (μg C CH_4_/CO_2_ m^−2^ h^−1^)
S: Time derivative (slope) CH_4_ and CO_2_ concentration change over time (ppm s^−1^)
V: Chamber volume (m^3^)
A: Chamber area (m^2^)
M: Molecular mass of CH_4_/CO_2_ (g mol^−1^)
V_m_: Ideal gas mole volume (0.0224 m^3^ mol^−1^)

Each regression plot was individually assessed and their R-squared values were used as a form of quality control for the selection of fluxes incorporated into the analysis; 94 % of all fluxes had a R-squared value of 0.7 or above (of which, 83 % had a R-squared value of 0.9 or above).

### Environmental measurements

Measurement of environmental variables (thaw depth, water table height) and soil parameters (pH, temperature and moisture) were performed at the same time as flux sampling in each plot. Soil temperature was measured just below the soil surface (1–4 cm) and at depth (9–11 cm) using a portable type T thermocouple, volumetric soil moisture was measured within the top 20 cm of soil (TDR 300 Fieldscout, Spectrum technologies INC) and soil pH at 3–7 cm (Thermo Scientific Orion 3-Star Plus pH Meter). The pH probe was calibrated against standards (pH 3 and 7) before starting the field campaign, and regularly during the field season, as a quality control of the measurements. Thaw depth and water table height were measured using a graduated metal rod, as described in Zona et al. (2009). Ambient air temperatures were recorded by the LGR™ Ultra-Portable Greenhouse Gas Analyser. Percentage vascular plant and moss cover was estimated visually after the end of the field season using photographs collected from each plot, during each sampling week.

### Statistical analysis

The importance of the variables explaining CH_4_ fluxes was determined using linear mixed models. CH_4_ fluxes were log transformed to meet the normality and homoscedasticity assumptions required for the analyses. All statistical analyses were carried out in R version 3.1.0 (R Core Team 2014). The following variables, their two-way interactions and squared terms were all tested as candidate explanatory variables; ER, NEE, GPP, thaw depth, water table depth, soil temperature at 9–11 cm, soil moisture, soil pH and percentage vascular plant cover. Initially, curvature in the relationship between explanatory and response variables was tested by fitting all explanatory variables and their squared terms, and only those statistically significant quadratic terms were retained. A series of models each containing main effects and a subset of all possible two way interactions were used to identify potentially significant interactions. A full model was then constructed using all main effect terms plus the quadratic and two way interactions already identified by the procedure described above. This model was simplified by the sequential removal of non-significant terms until removal of further terms caused an increase in AIC (Crawley 2012). For all mixed models the identity of the chamber (chamber ID) was included as a random intercept term to account for the repeated measurements taken at the same plots. Interactions were interpreted using the methods of Aiken and West (1991). Marginal R^2^ (R^2^_LMM(m)_), which describes the proportion of the variance in the data explained by the fixed effects, and conditional R^2^ (R^2^_LMM(C)_) which describes the proportion of the data explained by both fixed and random effects were calculated following Nakagawa and Schielzeth (2013). Model fits were checked visually to ensure that they conformed to model assumptions. Final *p* values were Bonferroni adjusted (multiplied by 54, the number of candidate explanatory variables) to mitigate the risk of type I error.

Because missing data for some variables (e.g., soil moisture data were missing due to power failure of the instrument) limited the number of observations available for the multiple regression, further mixed effect models were used to assess the importance of percent vascular plant cover and water table height on CH_4_ flux. Vascular plant cover and water table (above/below surface) were included as fixed effects and chamber was included as a random intercept. Initially three levels of the vascular plant cover were included (<10, 10–60 and >60 %) however this was reduced to two levels (<10 and >10 %) following model simplification. Further mixed effect models were fitted to test the impact of soil submergence on ER, NEE, GPP and CH_4_ flux. In each of these models, submergence (water table above/below soil surface) was fitted as a fixed effect while chamber ID was included as a random intercept. The dependant variable was transformed where necessary to meet the assumptions of homoscedasticity and homogeneity of variance.

As the sampling plots were stratified by wetness, we also tested the difference in NEE, ER, GPP, and CH_4_, between dry and wet sites by using a mixed model, again with chamber included as a random intercept. Wald test *p* values are presented.

## Results

### Environmental variables

**Fig. 2:**
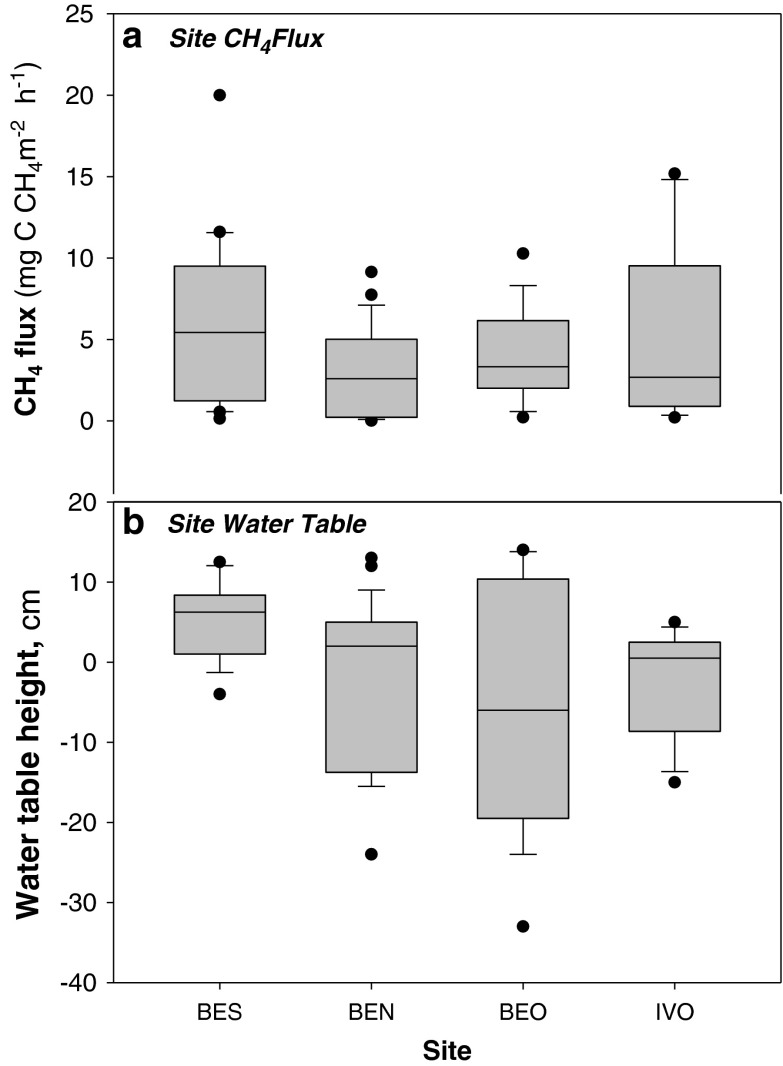
a) CH_4_ flux (mg C CH_4_ m^−2^ h^−1^) in August in BES (fairly flat, homogenous terrain) (n = 20, 1–25 August), BEN (low-centre, developed polygons) (n = 24, 1–25 August), BEO (high-centre, developed polygons) (n = 18, 6–26 August) in Barrow and Ivotuk (no substantial polygon development) (n = 11, on 18 August) and b) ground water table, cm, at sites BES (n = 18), BEN (n = 30), BEO (n = 20) in Barrow and Ivotuk (n = 12). Boxplots represent median (*midline*), quartiles (*box*), maximum and minimum (*whisker*) with outliers represented as *black points*

During the course of the experiment, average air temperatures in Barrow and Ivotuk were 10.9 ° C ± 5.36 s.d. and 5.6 ° C ± 0.32 s.d. respectively, with peak temperatures in Barrow in early August (max. 21.8 ° C) decreasing steadily throughout August and September (min. 1.38 ° C). Thaw depths in Barrow ranged from 25 to 47 cm below the surface in wet sites (average of 34 cm ± 4.04 s.d., n = 62) and from 10 to 42 cm below surface in the dry sites (average of 34 cm ± 6.68 s.d., n = 48) and in Ivotuk from 44 to 53 cm below surface in wet sites (average of 48 cm ± 3.25 s.d., n = 7) and from 45 to 50 cm below surface in dry sites (average of 48 cm ± 2.11 s.d., n = 5). Water tables within wet plots ranged from surface to 16 cm above surface (average 7 cm ± 11.5 s.d., n = 62) at Barrow and from surface to 5 cm above surface (average 2 cm ± 6.4 s.d., n = 7) in Ivotuk (Fig. 2). Water tables within dry plots in Barrow ranged from 1 to 33 cm below surface (average 13 cm ± 11.6 s.d., n = 44) and from 8 to 15 cm below surface (average 9 cm ± 5.2 s.d., n = 5) in Ivotuk. Across all sites, surface soil temperature (1–4 cm) ranged from 0.2 ° C to 14.6 ° C (average 5.9 ° C ± 4.0 s.d., n–122) and deeper soil temperatures (9–11 cm) ranged from 0.3 to 9.1 ° C (average 3.8 ° C ± 2.5 s.d., n = 122). Soil pH was consistently acidic, ranging from 2.7 to 6.5 (average 4.4 ± 0.7 s.d., n = 94) throughout the measurement period. Thaw depth was weakly correlated to both soil temperature (R^2^ = 0.08) and water table (R^2^ = 0.06) within wet sites, where wetter and warmer soils tended to have deeper thaw.

### Spatial variability in and influence of water table depth on CH4 fluxes

**Fig. 3:**
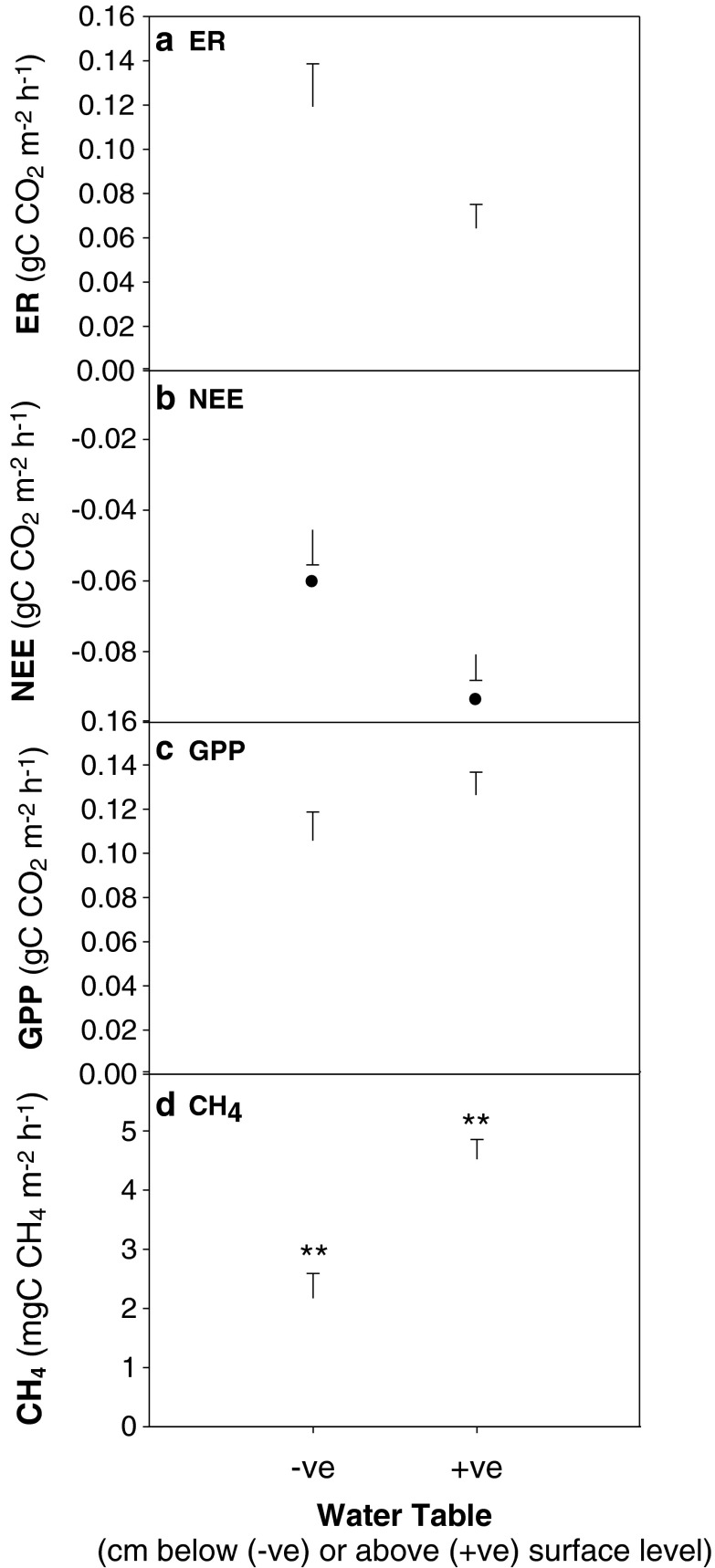
Average **a**) Ecosystem Respiration **b**) Net Ecosystem Exchange and **c**) Gross Primary Production fluxes (g C CO_2_ m^−2^ h^−1^) and d) CH_4_ flux (mg C CH_4_ m^−2^ h^−1^) at sample locations in Barrow (BES, BEO and BEN) and Ivotuk split by sites where water table height is below (-ve) or above (+ve) surface level (cm). *Bars* represent means with *error bars* shown as standard errors. ** denotes bars are significantly different at *p* < 0.01, ● denotes *p* < 0.1

CH_4_ emission was observed across all sites with no CH_4_ uptake recorded even in the driest of plots. High variability in CH_4_ emission was recorded, with rates ranging from 20 mg C CH_4_ m^−2^ h^−1^ (measured on the 10/08/2013 in Barrow) to 0.01 mg C CH_4_ m^−2^ h^−1^ (measured on the 11/09/2013 in Barrow) (Fig. 2), corresponding with decreasing air temperatures from 21.2 ° C (Barrow, 10/08/2013) to 7.7 ° C (Barrow, 11/09/2013). As expected, the wettest site (BES) showed the highest CH_4_ emissions (Fig. 2 and Fig. 3). The average of the entire measurement period indicated that CH_4_ emissions were significantly greater from wet sites (4.52 mg C CH_4_ m^−2^ h^−1^ ± 0.45 s.e., n = 64) compared to dry sites (2.17 mg C CH_4_ m^−2^ h^−1^ ± 0.55 s.e., n = 42) (Wald test, n = 106, F 1,75 = 8.2, *p* = 0.005) (Fig. 3d). The spatial variability in water table heights was more pronounced in the sites with more developed polygons (BEO: high centre polygons and BEN: low centre polygons; Fig. 2b). However, this variability in water table levels was not reflected in a similar variability in CH_4_ fluxes, which were more variable in the BES and Ivotuk sites despite their lower degrees of polygonization (Fig. 2a).

### The influence of water table depth on CO2 fluxes

There was a marginally significant trend for net ecosystem exchange (NEE) to be more negative (i.e., more net ecosystem CO_2_ uptake) in wet sites (-0.08 g C CO_2_ m^−2^ h^−1^ ± 0.1 s.e., n = 51) compared to dry sites (−0.05 g C CO_2_ m^−2^ h^−1^ ± 0.01 s.e., n = 36; Wald test, n = 87, F1,67 = 3.552, *p* = 0.0638; Fig. 3b). However ER (Wald test, n = 93, F1,61 = 0.628, *p* = 0.4309; Fig. 3a) and GPP (Wald test, n = 72, F1,52 = 0.972, *p* = 0.3287; Fig. 3c) did not differ significantly between the wet and dry sites.

### Environmental and vegetation control on CH4 flux

Based on our multiple regression modelling, the most important variables explaining CH_4_ fluxes were GPP and water table depth, followed by the interaction between water table and soil temperature (Table 1). All these variables combined explained 60 % (R^2^_LMM(m)_ = 0.60) of the variability in CH_4_ fluxes across the four sites investigated (Table 1).

**Table 1:** Parameter estimates for the fixed effects in a linear mixed model of the variables influencing CH_4_ flux; *n* = 51, R^2^_LMM(m)_ = 0.60, R^2^_LMM(c)_ = 0.77. Bonferroni adjusted *p* values are displayed

Parameter	Estimate	SE	df	*t*	*p*
Intercept	3.750942	0.525874	28	7.132776	<0.001
GPP	19.017379	4.478884	28	4.246008	0.011
Water table depth	0.170248	0.043746	28	3.891735	0.032
Soil temperature at 9–11 cm depth	0.153864	0.165532	28	0.929509	1.000
pH	−0.388229	0.781263	28	−0.496925	1.000
pH^2^	−1.585871	0.588373	28	−2.695347	0.637
Water table depth* Soil temperature at 9–11 cm depth	0.067599	0.015570	28	4.341595	0.011

Methane flux increased with increasing GPP (Table 1). GPP was significantly higher when vascular plant cover was >10 % in comparison to <10 %, and this relationship explains 18 % of the variation in GPP (mixed effect model, p = 0.005, R^2^_LLM(m)_ = 0.176, R^2^_LMM(c)_ = 0.176) while soil temperature (at 9–11 cm depth) explained 43 % of the variation in GPP (mixed effect model, p < 0.001, R^2^_LMM(m)_ = 0.431, R^2^_LMM(c)_ = 0.622)

**Fig. 4:**
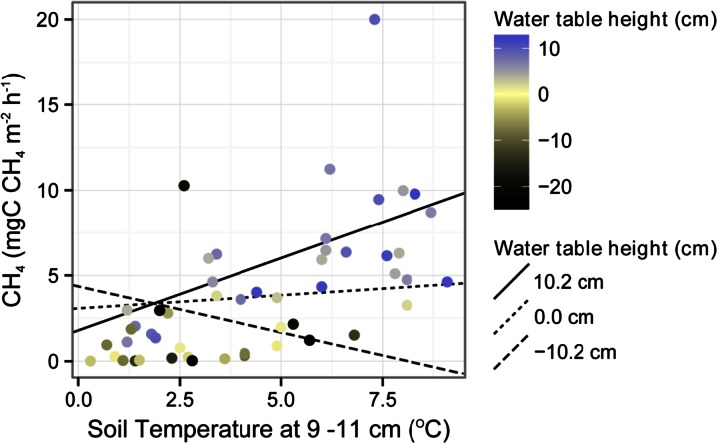
The influence of the interaction between soil temperature 9–11 cm below the surface and water table depth on CH_4_ flux. Points are mapped onto a colour scale to show the water table depth for each measurement. Regression lines show conditional influence of soil temperature on CH_4_ flux at the mean water table height (0.0 cm above the surface) and at 1 standard deviation above and below the mean (10.2 and −10.2 cm respectively) determined using the methods of Aiken and West (1991). For statistics see Table 1

There was a conditional effect of water table depth on CH_4_ emissions, with those sites with a deeper water table being more conducive to CH_4_ emission (Table 1, Fig. 3). This conditional effect was influenced by a significant interaction between water table depth and soil temperature at 9–11 cm (Table 1, Fig. 4). As the depth of the water table increased, the relationship between CH_4_ emission and soil temperature switched from negative to positive, with the sign of the slope of the relationship changing near the point where the water table is just above the soil surface (Fig. 4).

**Fig. 5:**
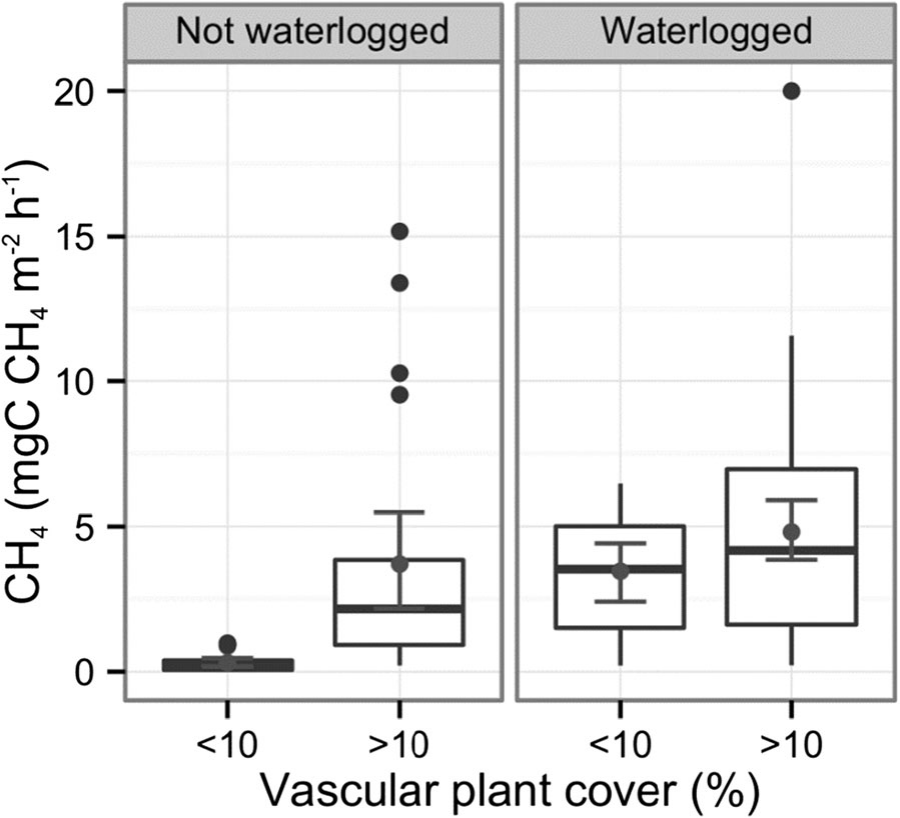
The influence of water table depth (above or below the soil surface) and vegetation cover on CH_4_ flux. Boxplots represent median (*midline*), quartiles (*box*), maximum and minimum (*whisker*) with outliers represented as black points. Grey points with error bars represent means with 95 % bootstrapped confidence intervals. For statistics see Table 2.

Methane emmissions were influenced by a significant interaction between soil wetness (water table above ground surface vs. below ground suface) and percentage vascular plant cover (Table 2, Fig. 5). Importantly, within wet sites, CH_4_ emissions were less dependent on vascular plant cover (increasing from 3.3 to 4.69 mg C CH_4_ m^−2^ h^−1^), whereas in dry sites there was a much more substantial increase in CH_4_ emission (almost an order of magnitude) from 0.35 mg C CH_4_ m^−2^ h^−1^ (n = 29) to 2.45 mg C CH_4_ m^−2^ h^−1^ (n = 30) with increasing vascular plant cover (Fig. 5, Table 2). Dry sites with >10 % vascular coverage had an average CH_4_ emission (2.46 mg C CH_4_ m^−2^ h^−1^, n = 30) similar to that in wet sites with <10 % vascular cover (3.29 mg C CH_4_ m^−2^ h^−1^, n = 27) (Fig. 5). The combination of soil wetness and vegetation cover explained 56 % of the variation seen in CH_4_ emissions (R^2^_LMM(c)_ = 0.56, Table 2).

**Table 2:** Parameter estimates for the fixed effects in the linear mixed effects model of the effect of waterlogging and degree of vascular plant cover on CH_4_ flux; *n*=108, R^2^_LMM(m)_ = 0.52, R^2^_LMM(c)_ = 0.56

Parameter	Estimate	SE	df	*t*	*p*
Intercept	−1.89	0.258	74	−7.34	<0.001
Vascular plant cover	2.59	0.347	28	7.47	<0.001
Waterlogging	2.81	0.396	74	7.09	<0.001
Vascular plant cover*waterlogging	−2.29	0.486	74	−4.71	<0.001

## Discussion 

All sites, representing a diversity of conditions given the high spatial heterogeneity, had positive CH_4_ flux across the entire experimental period, even the driest sites (water table about 24 cm below the surface) had relatively low emissions of <1.5 mg m^-2^ h^−1^. This is in contrast to some previous studies that have found CH_4_ uptake in dry soils due to oxic layers reducing CH_4_ production while promoting oxidation (Chen et al. 2014; Whalen and Reeburgh 1990). This was probably due to the substantial CH_4_ emission rates that occur during the growing season, in this nutrient rich, anaerobic environment, which is favourable to high rates of methanogenesis (Christensen et al. 2002; Grunfeld and Brix 1999; Harazono et al. 2006; Mastepanov et al. 2013; Morrissey and Livingston 1992; Sturtevant and Oechel 2013).

**Fig 6:**
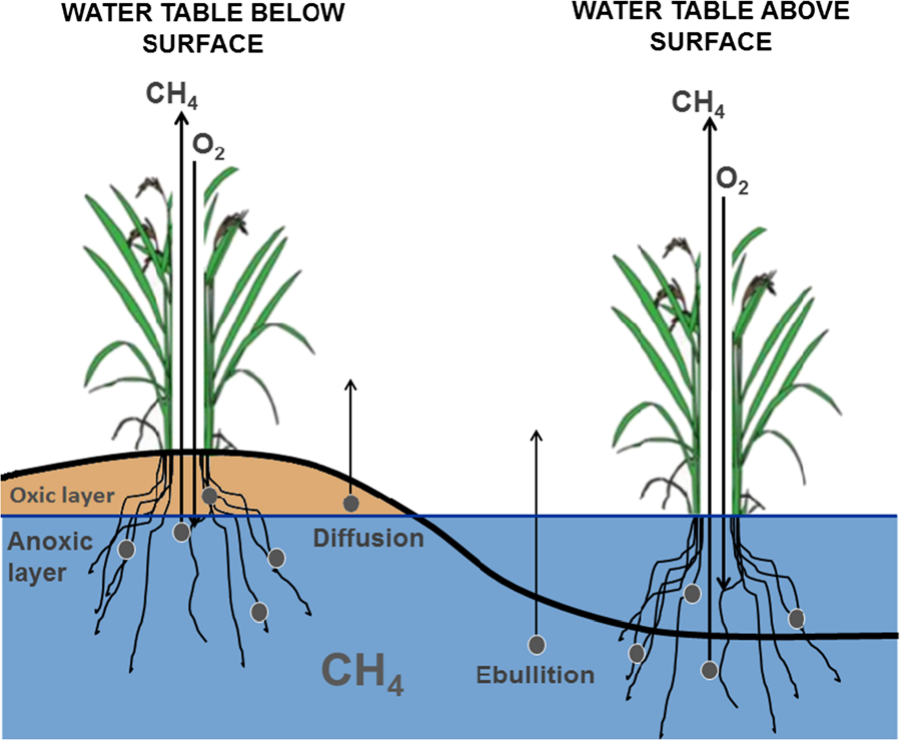
CH_4_ exchange within arctic tundra. CH_4_ is transported to the atmosphere directly through diffusion from the soil and indirectly through the roots and stems of vascular plants. In opposition, CH_4_ oxidation is aided by O_2_ diffusion directly into the soil and root aeration

The most significant control on CH_4_ fluxes across all the sites was found to be GPP. This may suggest a dominant role of plant productivity on CH_4_ emissions, as higher plant productivity (i.e., higher GPP) is likely to stimulate CH_4_ emission by providing photosynthetically derived substrates for methanogenic processes (Harazono et al. 2006; Lai et al. 2014b). However those plots with the highest GPP also tended to have a greater percentage cover of vascular plants, meaning both substrate input and the provision of CH_4_ transport pathways may have increased simultaneously (Lai et al. 2014b; Shannon et al. 1996; Fig. 6). In comparison to mosses, vascular plants have a higher photosynthetic capacity and their substantial root exudation and litter input increase substrate availability for methane production (Olivas et al. 2011; Riutta et al. 2007). Furthermore, vascular plants play a critical role in the transport of CH_4_ from the soil (Joabsson et al. 1999; Noyce et al. 2014), which is a key limit on CH_4_ flux, where emissions can depend more on the transport than CH_4_ production itself (Born et al. 1990; Harazono et al. 2006). With an absence of vascular plants, within drier sites at the polygon rims, limitation of transport and/or substrate availability appeared to be of major relevance in suppressing CH_4_ emission to relatively low levels (Fig. 5 and Fig. 6). For this reason, very low CH_4_ emissions were observed with low vascular plant cover (<10 %) within dry oxic sites (Fig. 5) in comparison to wet sites at the polygon centre, where CH_4_ can diffuse directly from the surface water (Fig. 6). However, in sites with vascular plants present, CH_4_ was transported through plant stems, bypassing oxic soil layers where it would otherwise be re-oxidised by methanotrophs (Joabsson and Christensen 2001; Shannon et al. 1996; Fig. 6). Mechanistically, vascular plants act as a conduit for methanogenesis, connecting the CH_4_ produced at depth within the soil to the atmosphere, thereby enhancing the release of CH_4_ (von Fischer et al. 2010; Harazono et al. 2006; Joabsson and Christensen 2001; Sebacher et al. 1985; Shannon et al. 1996).

The ability of vascular plants to both transport CH_4_ and provide soil C for methanogenesis varies by species. For example, the presence of *Eriophorum ssp* (cotton grass) results in CH_4_ emissions between 1.4–2.2 and 3.7–5.5 times higher than the *Maianthemum/Ledum* and the shrub *Chamaedaphne* communities respectively (Lai et al. 2014a). The amount and extent of plant roots varies between vascular species, where deeper and wider root structures facilitate the increased production and release of CH_4_ from soil layers below the water table and closer to the permafrost layer (Harazono et al. 2006; Joabsson and Christensen 2001; Lai et al. 2014a; Shannon et al. 1996). However, we show in this study that the influence of vegetation on CH_4_ emissions is strongly dependent on the water level and this interaction must be taken into account when considering overall CH_4_ loss. With sparse vascular plant cover, wet sites tend to be higher CH_4_ emitters than dry sites (Fig. 5). On the other hand, in the presence of substantial vascular plant cover, both wet polygon troughs and dry oxic rims emitted substantial CH_4_ (Table 2, Fig. 5). This created local scale spatial variability within the ice wedge polygon landscape in relation to vascular plant community cover. It should be mentioned, however, that downward transportation of O_2_ into the soil by vascular plants can increase methane oxidation by methanotrophs, lowering CH_4_ emission (Frenzel and Rudolph 1998; Harazono et al. 2006; Heilman and Carlton 2001; Ström et al. 2005). This process, however, was likely to be less important in comparison to the enhancement of both CH_4_ transport and carbon (C) supply, and never resulted in an uptake of CH_4_, even within the driest sites during this study (Fig. 5). NEE was marginally significantly lower in the wetter sites, perhaps because plant productivity was promoted due to increased nutrient availability resulting from warmer temperatures in these soils (Nadelhoffer et al. 1991; Rustad et al. 2001). Hence the wet conditions which promote CH_4_ emission by causing anoxia may further promote CH_4_ emission by increasing GPP and vascular plant growth, which could promote both CH_4_ transport and substrate production (Joabsson et al. 1999; King et al. 2002; Rinnan et al. 2007; Ström et al. 2003).

Water table depth was the next most significant control on CH_4_ emission after GPP, with wet sites showing higher CH_4_ emission (Table 1, Fig. 3). This is consistent with other studies where site wetness has been found to be a strong driver of CH_4_ emission due to the high abundance of methanogens in anaerobic, waterlogged conditions (Bubier et al. 1993; von Fischer et al. 2010; Lai et al. 2014a; Moore and Roulet 1993; Roulet et al. 1992; Zona et al. 2009). Christensen et al. (2003) described water table as an ‘on-off switch’ controlling CH_4_ flux, while other factors control CH_4_ flux within water tables shallower than a certain threshold, above which site wetness governs CH_4_ emission. On the other hand, wet sites are not always found to be correlated with higher CH_4_ emission, for example Brown et al. (2014) found a critical zone for maximum rates of methanogenesis at 40 to 55 cm below the surface, which they speculated coincided with the maximum provision of fresh organic material and necessary redox potentials, in addition to facilitating the potential degassing of stored CH_4_. In our sites the water table was never below 33 cm, which may have explained the substantial CH_4_ losses in all of the sites measured here, including the driest (Fig. 5).

Interestingly, water table level determined the temperature dependence of CH_4_ emissions, as shown by the significant interaction of water table and soil temperatures on CH_4_ loss (Table 1, Fig. 4). Wetter peat soils tend to be warmer due to a higher heat capacity of water (Dunfield et al. 1993; Whiting and Chanton 1993). Generally, higher soil temperatures are expected to increase substrate availability and the abundance of methanogens in peat, and therefore CH_4_ emissions (Dunfield et al. 1993; Valentine et al. 1994). Increases in temperatures from 2 to 12 ° C have been correlated with an increase in CH_4_ emission by a factor of 6.7 (von Fischer et al. 2010; Svensson and Rosswall 1984). However, in the dry, oxic sites, CH_4_ oxidation occurs together with methanogenesis, and these two processes might cancel each other out resulting in the lack of a net increase in CH_4_ emissions with temperature increase (Lai et al. 2014a; Svensson and Rosswall 1984; Zhu et al. 2014). This result suggests the need to stratify the measurements in this highly polygonized tundra environment to be able to capture the different response of different microtopographic features, including both dry and wet sites.

In addition to water table, thaw depth has been found in other studies to be a key control on CH_4_ emission from tundra ecosystems (Nakano et al. 2000; Sturtevant and Oechel 2013; Verville et al. 1998; Zona et al. 2009). However, there was fairly low variability in thaw depths in this study (from 26 to 42 cm below surface level), partially because of the limited temporal range of sampling (from peak to late season) across sites, and this may have explained why it was not found to be significant in explaining CH_4_ fluxes. This contrasts with previous work within this region (Harazono et al. 2006; Morrissey and Livingston 1992; Torn and Chapin 1993; Zona et al. 2009, 2011) which showed thaw depth to be a critical control of fluxes over the growing season (but these studies included early as well as late season, resulting in a broader range of thaw depths). Within this acidic tundra, pH across the study sites presented a large variation (2.7–6.49) and yet did not significantly correlate with CH_4_ fluxes, however the highest CH_4_ emissions were observed at a pH of around 4.2. These unusually low pH values (2.7–3.4) were found in Ivotuk plot sites, where similar values (down to 2) have been previously recorded within a similar ecosystem (Lipson et al. 2012). Due to the particularly dry conditions, dry plot sites with low pH were probably more oxidised than usual (for example oxidation of Fe(II), S compounds and NH_4_^+^) releasing protons and making these extreme soil pH values possible within localised areas of the tundra (Lipson, personal communication). In contrast, the few wet sites found with low pH had high proportions of peat accumulation and dense moss cover, mostly characterised by dwarf shrub and acidophilic mosses that further secrete organic acids during growth (Gornall et al. 2007; Hobbie and Gough 2004; Riedel et al. 2005). Variable responses of CH_4_ emissions on soil pH have been previously reported in field studies ranging from no correlation (Brummell et al. 2012; Ohtsuka et al. 2006), to positive correlations (Moore et al. 1990) and negative correlations (Kato et al. 2011; Walker et al. 1998).

In our study, CH_4_ fluxes ranged from 0.01 to 20 mg C CH_4_ across a spatially dynamic environment, with wetter sites with higher GPP having higher emission. This high spatial variability, where CH_4_ emissions can vary by an order of magnitude between different plots, has consistently been found across other studies where daily averages can range some 4.5 to 9.6 mg C CH_4_ m^−2^ d^−1^, across consecutive measurements within the same sites (Harazono et al. 2006; Schimel 1995; Shannon et al. 1996; Wille et al. 2008). The general scarcity of data on the plot scale from these arctic environments limits our understanding of the controls over this large variability in CH_4_ fluxes, where fine scale datasets are critical for increasing our understanding of the smaller scale landscape heterogeneity (Sachs et al. 2010). Fine-scale relationships between CH_4_ fluxes, vegetation and environmental conditions might be missed by eddy covariance measurements measuring C fluxes over a wider scale in these highly heterogeneous arctic ecosystems (Fox et al. 2008; Sachs et al. 2010; Wickland et al. 2006). Therefore our results, as measured by chambers, might prove very useful for identifying the detailed relationship between environmental and vegetation controls, namely GPP, water table depth and soil temperature, and for describing how fluxes relate to fine scale microtography.

## Conclusion

In this study we showed that multiple complex processes driving CH_4_ flux emissions within the wet sedge and tussock tundra ecosystems interacted with each other in controlling CH_4_ flux. Crucially we have demonstrated the importance of vascular plant cover in determining CH_4_ flux and that increased vascular plant cover can promote CH_4_ production and release from both waterlogged and drier soils. The most important environmental control on CH_4_ emissions within our study locations was GPP. Vascular plant coverage seemed to be the factor most correlated with CH_4_ emissions within dry sites, highlighting the importance of CH_4_ oxidation and potentially labile C availability in controlling emissions from the high-centre polygons and rims. In these dry sites, greater vascular plant cover increased CH_4_ emission by almost an order of magnitude to levels equivalent of wet sites. Overall, given the importance of vascular plant cover on CH_4_ emissions, hydrological changes in the Arctic might affect CH_4_ emissions very differently depending on the plant communities present and how they develop under a changing climate.
